# *Leptospira* Seroprevalence in Colombian Dairy Herds

**DOI:** 10.3390/ani11030785

**Published:** 2021-03-11

**Authors:** Simone Taddei, Giovanni Moreno, Clotilde Silvia Cabassi, Emiliana Schiano, Costanza Spadini, Sandro Cavirani

**Affiliations:** 1 Department of Veterinary Science, University of Parma, Via del Taglio 10, 43126 Parma, Italy; clotildesilvia.cabassi@unipr.it (C.S.C.); emiliana.schiano@unipr.it (E.S.); costanza.spadini@unipr.it (C.S.); sandro.cavirani@unipr.it (S.C.); 2 Facultad de Ciencias Agrarias, Fundación Universitaria Juan de Castellanos, Carrera 11 # 11-44, Tunja 150001, Boyacá, Colombia; gmorenof@jdc.edu.co

**Keywords:** leptospirosis, bovine, dairy, seroprevalence, Colombia

## Abstract

**Simple Summary:**

Leptospirosis is one of the waterborne diseases whose spread could be significantly affected by global changes that act on the environment, both in Latin America and in other parts of the world. However, there are few studies regarding leptospirosis in cattle from Latin America, especially from Colombia. The aim of the present study was to determine the overall and within-herd seroprevalence and mapping of different *Leptospira* serovars in dairy cattle from farms located in some municipalities of the Colombian department of Boyacá. A high proportion (95%) of herds with at least one seropositive animal was found. Moreover, within-herd seroprevalence was very high in 20% of the herds. The frequent presence of other domestic animals on farms could be a risk factor for the spread of the infection. Human leptospirosis seroprevalence in some areas of Colombia is high. However, we found that the most common serotypes involved in human disease were the ones with the lowest seroprevalences in cattle in the investigated area. This suggests that cattle could represent a minor risk factor for the transmission of *Leptospira* infection to humans. Nevertheless, the need for stricter preventive measures in cattle farms has emerged.

**Abstract:**

Leptospirosis in cattle has important economic effects on the infected farms. Moreover, livestock farming is considered a major occupational risk factor for the transmission of *Leptospira* infection to humans. A survey was performed to determine the overall and within-herd seroprevalence and mapping of different *Leptospira* serovars in dairy cattle from farms located in some municipalities of the Colombian department of Boyacá. Nine hundred and fifty-nine animals, from 20 unvaccinated and one vaccinated herd, were included in the study. Anti-*Leptospira* serum antibodies were detected by the microscopic agglutination test (MAT). Only one herd was seronegative. Overall seroprevalence to at least one serovar of *Leptospira* was 24.1% for unvaccinated animals and 62.3% for animals from the vaccinated herd. A very high within-herd seroprevalence (>60%) was present in 20% of the unvaccinated herds. The presence in the vaccinated herd of 20/398 animals showing high titers, between 1000 and 4000, to at least one serovar of *Leptospira* suggest that some animals could have been infected. Moreover, due to the presence of seronegative animals, a failure of vaccination immunity or the presence of unvaccinated animals in the vaccinated herd cannot be excluded. In all farms, domestic animals other than cattle were present. Considering the farming practices occurring on dairy farms in the study area, higher hygienic standards and stricter biosecurity measures are suggested.

## 1. Introduction

Leptospirosis is a zoonotic infectious and contagious disease transmitted mainly via direct contact with the carrier’s urine or indirectly through a urine-contaminated environment. The products of abortion in domestic animal species may also be a route of transmission of the infection [1]. The spread of the infection is facilitated by the fact that the infection can be asymptomatic or paucisymptomatic, especially when the infected host is the reservoir of the bacterium. Survival outside the host largely depends on humid and warm conditions [1]. Leptospirosis is one of the waterborne diseases whose spread could be significantly affected by global changes that act on the environment, primarily climate changes, both in Latin America than in other parts of the world [1,2,3]. Leptospirosis incidence in tropical and developing countries is generally higher than those in temperate and developed countries, due to favorable climatic conditions and lower hygienic measures [3,4,5,6,7]. Moreover, closer contact with animals, which usually occurs in developing countries, has a positive impact on zoonotic transmission of the infection [8]. Tropical and subtropical Latin American ecology is characterized by abundant rainfall and by the presence of many natural water courses, as well as by high temperatures, which are favorable conditions for the transmission of leptospires [3]. In Colombia, the prevalence of *Leptospira* spp. antibodies in humans is high and comparable with reports from other Latin American countries, both in rural and urban environments [3,9,10,11]. Moreover, human seroprevalence was found to be associated with exposure to animals and to rural social level [10]. Bovine leptospirosis is caused mainly by serogroup Sejroe serovar Hardjo and is widespread worldwide [12]. However, reliable estimates of serovar Hardjo infection prevalence, as well as accurate data for the frequency of abortion in cattle attributable to leptospirosis, are lacking [13]. Leptospirosis in cattle has important economic effects on the infected farms, resulting in reproductive losses due to infertility, abortions, stillbirths, weak offspring, and decreased milk production and growth rates. Moreover, livestock farming is a major occupational risk factor for the transmission of *Leptospira* infection to humans and a high risk is associated with dairy farms and with serovar Hardjo [14]. In addition to the type of breed (dairy vs beef), risk factors for cattle leptospirosis may include herd size, stocking density, and herd management—grazing in areas shared with other infected cattle, pig or sheep, presence of contaminated water sources, use of an infected bull, and age of the animals [5,6,15,16,17]. In Colombia, a very high overall seroprevalence was reported for dairy cattle in the north of Antioquia (equal to about 61%) and for dual-purpose cattle in rural areas of Ciénaga de Oro, Córdoba (74.5%) [18,19]. A 16.4% overall prevalence was instead reported for cattle in the municipality of Pereira [20]. To the authors’ knowledge, no data have been reported for cattle from the department of Boyacá, which is responsible for a large part of the Colombian dairy production. Despite an increase of interest in the last decade, there have been few studies regarding leptospirosis in cattle from Latin America, and this is especially true for Colombia [12].

The aim of the present study was to determine the overall and within-herd seroprevalence and mapping of different *Leptospira* serovars in dairy cattle from farms located in some municipalities of the Colombian department of Boyacá.

## 2. Materials and Methods

### 2.1. Study Sites

**Figure 1:**
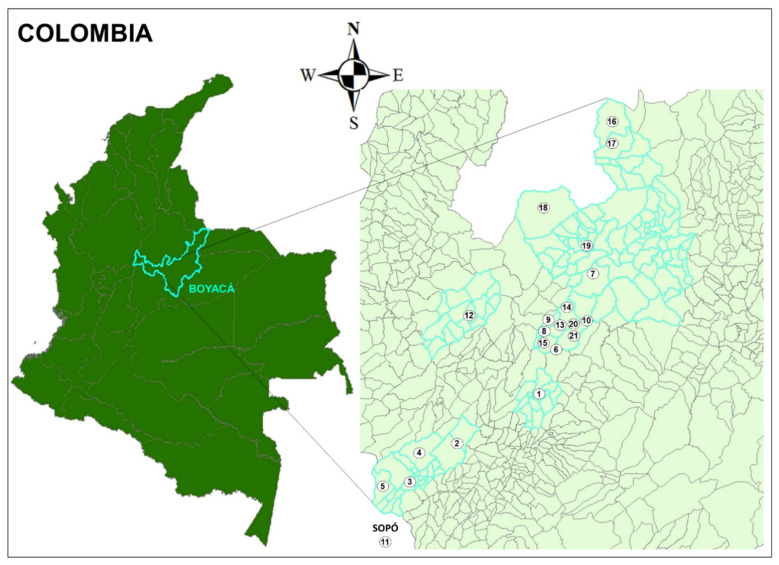
Map of study sites in Colombia. Farms are indicated by numbers, corresponding to those in Table 1.

Boyacá department lies in the center of Colombia, between the 04°39′10″ and 07°03′17″ north latitude and 71°57′49″ and 74°41′35″ west longitude. The department belongs to the Andean region and the east mountain range occupies most of the departmental territory. The sampling area, with the sole exception of Sopó municipality, belonging to the department of Cundinamarca, is included in the so-called “Cordón Lechero de Boyacá” (dairy area of Boyacá), within the Altiplano Cundiboyacense, and includes the municipalities of Ventaquemada, Oicatá, Tuta, Sotaquira, Paipa, Soracá, Chiquiza, and Sopó (Figure 1). It has an altitude ranging from 2630 to 2860 m above sea level, a mean annual temperature ranging from about 8 to 20 °C, a mean annual rainfall ranging from 500 to 2500 mm, and a mean annual relative humidity value (period of measurements 1981–2010) ranging from 75% to 85% [21].

### 2.2. Sampled Population

The total dairy cattle population of the considered municipalities was 88,255 animals (n = 13,364 Ventaquemada, n = 4209 Oicatá, n = 15,455 Tuta, n = 15,500 Sotaquira, n = 18,100 Paipa, n = 5815 Soracá, n = 2131 Chiquiza, and n = 13,681 Sopó). Excluding Sopó, this bovine population was distributed in 6357 small (≤50 cows), 138 medium (51–100 cows), and 56 large (>100 cows) dairy farms [22]. The study population comprised 959 animals belonging to Red Holstein, Black Holstein, and Normande breeds, or crosses between them. The study was conducted using blood serum samples collected from autumn 2016 until spring 2017 for a serological survey in Colombian dairy herds. No ethical approval was required because the study did not involve a prospective evaluation, did not involve laboratory animals, and only involved routine diagnostic procedures, commonly performed in bovine herds. This also applied to the collection of the samples for the previous serological survey. In particular, the study fell within the cases excluded from the scope of the national law Decreto Legislativo n. 26/14 (art. 2), regarding the execution of Directive 2010/63/EU on the protection of animals used for scientific purposes. The study was carried out in 18 small dairy farms, two medium dairy farms, and one large dairy farm, selected by convenience (Table 1). The criteria for excluding a farm from the study were the owner’s opposition to the study, the presence of potential safety risks for the personnel involved in the collection of samples and data, and inability to reach/access the farm. Farm information was retrieved on the day of blood sampling by an informal interview with the farm veterinarian and owner and by the review of records. Each animal underwent a brief clinical examination at the time of blood collection. Information regarding the level of modernization of the farm (milking mode and farm management) was collected through the observations made during the visit to the farm.

With the exception of farm 11, all the animals present in the herds were sampled. In farm 11, 398/700 randomly selected animals were sampled. The minimum number of animals to be sampled in farm 11 (n_c_ =341) was established by the following formula: n = Z^2^[P(1 − P)]/D^2^, where n = sample size, Z = Student’s t-value (set to 2.58 for a desired confidence level equal to 0.99), P = assumed true prevalence (set to 0.5, which corresponded to the maximum size of the sample), D = desired precision (set to 0.05). The sample size was then adjusted with finite population correction according to the following formula: n_c_ = n/1 + (n/N), where n_c_ = corrected sample size, n = sample size, N = population size. In almost all farms (18/21), only female animals were present due to the wide use of artificial insemination. Four sampled bulls were present in 3 farms (1 bull in farms 5 and 8, and 2 bulls in farm 11). Farms were classified within three different levels of modernization: low, medium, and high. In farms with low modernization, calves are not separated from cows and each calf is normally suckled by its own mother, milking is carried out manually, and cowsheds are not present. In farms with medium modernization, calves are separated from cows and fed from livestock staff, milking is carried out by portable milking machine, and pasture rotation is performed. In farms with high modernization, cowsheds are present and cows are tie-stall housed, a milking room is also present. Due to the very homogeneous dairy production systems in the region, some characteristics of farming were similar between the different herds. In the study area, grazing systems were mostly characterized by rotation of meadows, thus alternating periods of occupation of pastures with rest periods. Predominant grasses in the meadows were kikuyo grass (*Penisetum clandestinum*), alternating with red and white clover (*Trifolium pratense* and *Trifolium repens*, respectively). However, in approximately eighty percent of the studied sites (herds with medium and high modernization), farmers supplemented animals with raw potatoes on a daily basis at the time of milking, thus replacing the use of concentrate. Regarding vaccine plans, in all herds, cattle were vaccinated against brucellosis and foot and mouth disease (FMD). In farms with higher modernization levels, it is common practice also to vaccinate against widespread cattle pathogens such as bovine parainfluenza-3 virus (BPIV-3), bovine herpesvirus-1 (BoHV-1), bovine viral diarrhea virus (BVDV), bovine respiratory syncytial virus (BRSV), and *Leptospira*. Therefore, herd 11 was vaccinated with Cattlemaster 4 (Zoetis, Parsippany, NJ, USA) and with Triangle 9 (Boehringer, Ingelheim, Germany).

### 2.3. Serology

Blood samples were collected by coccygeal venipuncture into vacutainer tubes (Becton Dickinson, Franklin Lakes, NJ, USA) without anticoagulant, kept at refrigeration temperature, and delivered to the laboratory within 24 h. Each serum was immediately separated by centrifugation at 1100× *g* and stored at −20 °C until it was analyzed. Anti-*Leptospira* serum antibodies were detected by the microscopic agglutination test (MAT). Suspensions of 7 strains of *Leptospira* (serogroup Sejroe, serovar Hardjo; serogroup Pomona, serovar Pomona; serogroup Australis, serovar Bratislava; serogroup Icterohaemorrhagiae, serovar Copenhageni; serogroup Grippotyphosa, serovar Grippotyphosa; serogroup Canicola, serovar Canicola; serogroup Tarassovi, serovar Tarassovi) were used as antigens. MAT was performed as previously reported [23]. Briefly, *Leptospira* strains were cultured in liquid EMJH medium (Becton Dickinson, Maryland, USA) to a density of approximately 2–4 × 10^8^ leptospires per ml and diluted 1:2 in sterile saline for the test. All sera were first screened at 1:100 dilution. Following incubation, each suspension was observed by a dark field microscope (Nikon, Eclipse 50i) at 100X magnification. Sera that gave a positive reaction were further titrated in serial twofold dilutions, starting from 1:125 to titer end-point. Antibody titers were expressed as the reciprocal of the highest dilution of serum that gave 50% or more of reduction of free leptospires in the suspension, compared to a negative control obtained by using sterile saline instead of serum. The presence of agglutination for all serovars tested would be considered to be caused by a nonspecific effect of the serum. A titer ≥100 was deemed positive, i.e., indicating exposure to *Leptospira*.

### 2.4. Statistical Analysis

Population data were compared with sample data to assess possible bias and determine whether the study sample represented the source population. For this purpose, average variability [24,25] was evaluated for the following variables: herd size and geographical location of the animals.

## 3. Results

**Figure 2:**
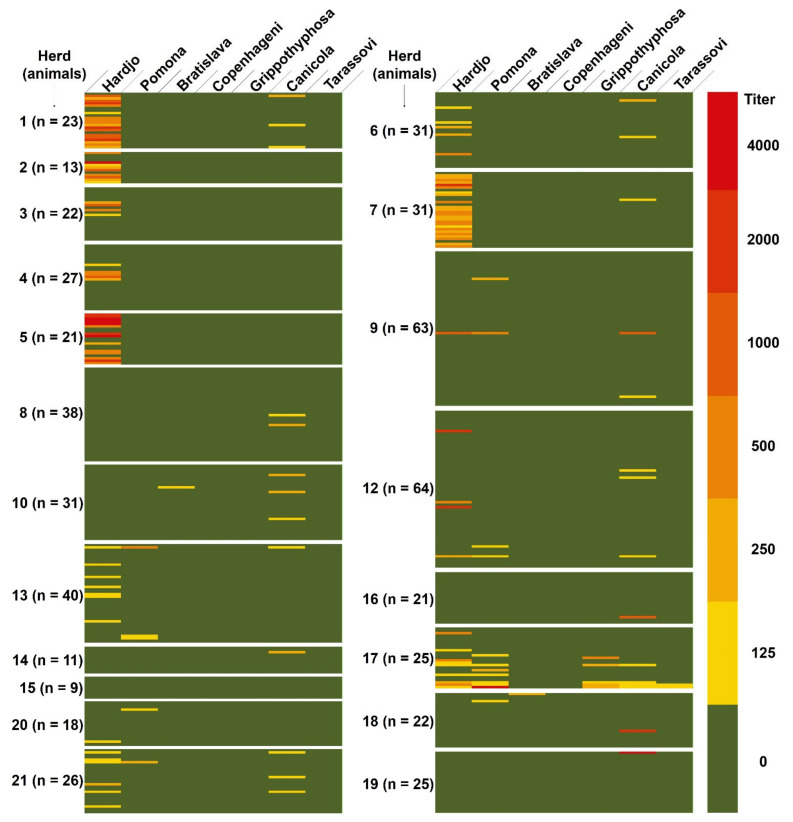
Heat map of microscopic agglutination test (MAT) seropositivity to the different serovars of *Leptospira* in unvaccinated herds.

**Figure 3:**
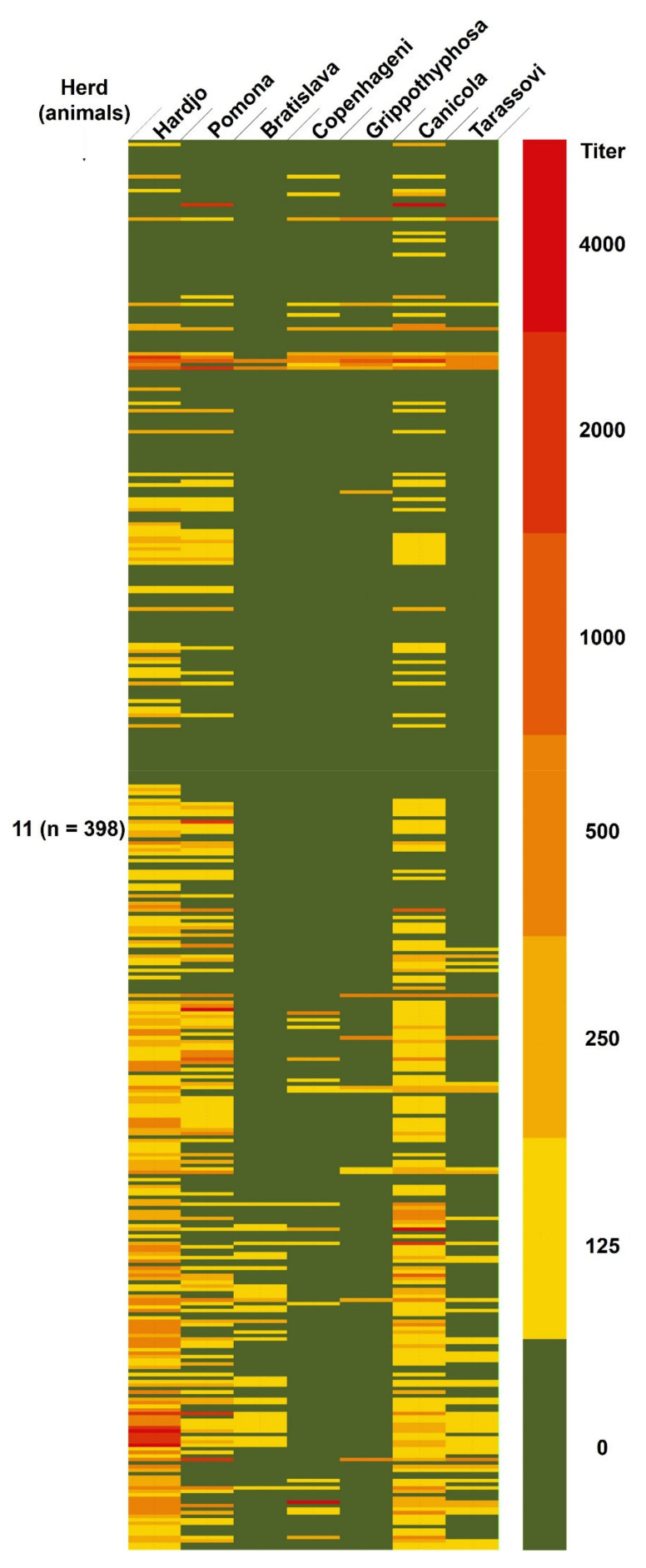
Heat map of MAT seropositivity to the different serovars of *Leptospira* in the vaccinated herd.

Farms with a higher level of modernization showed a more accurate record of anamnestic data and in some of the farms with a low level of modernization record keeping was very poor. Clinical evaluation of sampled animals was not included within the objectives of this study. However, from an examination carried out during sampling procedures, in no case was a clear clinical picture detected. Nevertheless, it is known that cattle usually present the reproductive form of the disease [12] and the presence of abortions was reported in 11/21 (52%) of the herds (Table 1). None of the sera reacted with all the tested strains of *Leptospira*, suggesting that none of the positive reactions should be considered nonspecific. Therefore, all the observed agglutinations were ascribed to specific antigen–antibodies complex formations. Moreover, all sera positive at the 1:100 screening dilution were confirmed positive at the 1:125 dilution. Seropositivities to different serovars in unvaccinated and vaccinated herds are shown in Figure 2 and Figure 3, respectively.

Overall seroprevalence, to at least one strain of *Leptospira*, was equal to 39.9% (383/959 animals). Considering the vaccination status, 135/561 (24.1%) of the unvaccinated animals and 248/398 (62.3%) of the animals from the vaccinated herd were seropositive to at least one serovar of *Leptospira*. Detailed overall seroprevalences, considering different serovars, are reported in Table 2. Some animals showed positivity to more serovars. Animals showing multiple positivities are reported in Table 3. The within-herd seroprevalence ranged from 0% to 83.9% for the unvaccinated herds and was equal to 62.3% (95% confidence interval = 59.1–65.5%) for the vaccinated herd (Table 4).

Twenty out of 398 animals from the vaccinated herd showed titers equal to or higher than 1000 to at least one serovar. In particular, 16 animals had a titer equal to or higher than 1000 against one serovar (four animals against Canicola, one animal against Copenhageni, seven animals against Hardjo, four animals against Pomona), three animals had a titer equal to or higher than 1000 against two serovars (one animal against Canicola and Pomona, two animals against Hardjo and Pomona), and one animal had a titer equal to or higher than 1000 against four serovars (Canicola, Grippotyphosa, Hardjo, and Pomona).

Source population data were compared with sample data to determine the representativeness of the convenience sample. Average variability was evaluated, with a 95% confidence interval under a normal approximation, for the following variables: herd size and geographical location of the animals. Ninety-five percent of the research sample’s count of small herds was expected to fall in the interval 20.38 ± 1.52, therefore small herds (n. = 18) were slightly underrepresented. Ninety-five percent of the research sample’s count of medium herds was expected to fall in the interval 0.44 ± 1.29, therefore medium herds (n. = 2) were slightly overrepresented. Large herds, although present in a low percentage in the considered area (0.85%), were underrepresented, because the large herd included in the study was vaccinated and this excluded it from the overall prevalence estimate of the study area. Regarding the geographic distribution of the source population, the research sample’s count of animals was in the acceptable range (100.53 ± 17.74) for the municipality of Ventaquemada (n. of animals = 83), Oicatá (n. = 298) was strongly overrepresented (acceptable range = 31.66 ± 10.67), and Chiquiza (n. = 64) was also overrepresented (16.03 ± 7.71), while Tuta (n. = 25; 116.26 ± 18.75), Sotaquira (n. = 22; 116.60 ± 18.77), Paipa (n. = 46; 136.16 ± 19.83), and Soracá (n. = 23; 43.74 ± 12.40) were underrepresented. Sopó was not considered, because from this municipality only the vaccinated herd was included in the study.

## 4. Discussion

A survey was performed to determine the overall and within-herd seroprevalence and mapping of different *Leptospira* serovars in dairy cattle from farms located in some municipalities of the Colombian department of Boyacá. Although in recent years a greater interest has emerged regarding bovine leptospirosis in Latin America, some areas of the continent, including Colombia, have not been sufficiently monitored and more information is required [3,12].

The overall seroprevalence evaluated on unvaccinated animals was equal to 24.1%. For the present study, this is the most likely estimate of the overall seroprevalence in the area of concern. In fact, it must be emphasized that the 39.9% overall apparent seroprevalence calculated on all animals was skewed by the presence of a large proportion of vaccinated animals. The overall seroprevalence value found here was much lower than those reported for cattle from the north of Antioquia (equal to about 61%) and the rural areas of Ciénaga de Oro, Córdoba (74.5%), but was relatively close to that reported for cattle in the municipality of Pereira (16.4%) [18,19,20].

Internal validity could be affected by test performance bias, also due to the possible presence of circulating serovars not used in the MAT. Two tests have a role in indirect veterinary diagnosis of leptospirosis: the MAT and the ELISA. The MAT is the most widely used serological test. It is the reference test against which all other serological tests are evaluated [26]. However, MAT is an imperfect test, having a sensitivity of less than 50% in some chronic infections, and for this reason, other methods to validate ELISAs have also been used [26]. The MAT cannot be standardized and is subject to wide variations in sensitivity, mostly depending on the antigens used in the test and the *Leptospira* serogroups existing in the region were the animals are found, but also on the test conditions [26]. For these reasons MAT performance is commonly not evaluated. As we did not adjust our prevalence results for test performance, they are actually apparent prevalence values.

In the present study, the number of sampled animals and selection of farms were not based on prevalence estimates and were not performed by random methods. The need to use a convenience sampling of herds was due to several reasons, including the ease of access, cooperation of the owner, personal safety, and a limited period of time for sampling. Therefore, though we tried to represent all productive districts of the considered municipalities, data obtained do not allow to have a bias-free picture of the overall seroprevalence in the area of concern.

The main limitation of the present study, affecting external validity, was the nonprobability sampling bias. Moreover, the overall low sample size did not allow to us assess possible associations between seropositivity and potential risk factors and data were therefore presented using descriptive statistics. The risk factors for bovine leptospirosis may vary widely in different parts of the world [27]. Different epidemiological studies into leptospirosis in beef and dairy herds have reported an association between herd size and herd- and/or animal-level seroprevalence for *Leptospira* infection in cattle [6,27,28]. Most of the studies reported a positive association between herd size and *Leptospira* seroprevalence. However, a negative association between herd size and *Leptospira* seroprevalence was reported for cattle in Colombian dairy herds [29]. No significant association between herd size and *Leptospira* seroprevalence was also reported [15]. In our case, small herds were slightly underrepresented, as well as large herds, whilst medium herds were slightly overrepresented. In consideration of the discordant indications deriving from the scientific literature regarding the effects of the herd size factor on *Leptospira* seroprevalence, it is difficult to assess in which direction the overall prevalence data were distorted by the herd size bias. Similarly, we had no data that allowed us to establish the direction and magnitude of the bias due to the geographical distribution of the sample with respect to the source population. Moreover, differences in susceptibility to *Leptospira* infection among different breeds of small ruminant have been reported [30,31]. Therefore, it cannot be excluded that also cattle breeds could be a source of bias. However, we have neither reliable scientific information on differences in susceptibility to *Leptospira* infection between the different cattle breeds nor data on the distribution of cattle breeds among the source population. Furthermore, the absence or infrequency of veterinary assistance was suggested to be positively poorly associated (OR = 1.74) with the overall seroprevalence to leptospirosis in cattle [16]. Veterinary assistance was present in all the sampled herds and this could also be a source of bias in the direction of an underestimation of the overall prevalence. The possible presence of other sources of biases affecting the overall prevalence estimate in different directions cannot be excluded.

However, as we carried out a survey on all the animals of the herds and not a sampling, with the exception of farm 11, our study, in addition to giving indications regarding the overall seroprevalence in the study area, is relevant relatively to the measurement of the within-herd seroprevalence. The within-herd seroprevalence ranged from 0% to 83.9% for the unvaccinated herds. In unvaccinated herds with a very high (>60%) within-herd seroprevalence (herds 1, 2, 5, 7), *Leptospira* Hardjo was almost completely responsible for total within-herd seroprevalence. Only in herd 7 did serovar Canicola contribute to increased within-herd seroprevalence due to the presence of one animal that was only positive to Canicola (Figure 2 and Table 4). In unvaccinated herds with a lower within-herd seroprevalence (<50%), herds 3 and 4 were positive only to *Leptospira* Hardjo, whilst in remaining herds also other serovars contributed to (herds 6, 8, 9, 12, 13, 17, 20, 21) or were responsible for (herds 10, 14, 16, 18, 19) the total within-herd seroprevalence (Figure 2 and Table 4). Only one herd with a low number of animals (herd 15) was completely seronegative.

Otte and colleagues [32] found the following overall prevalences of seropositivity for cattle from herds located in the department of Meta: Hardjo 45.9%, Canicola 6.7%, Pomona 4.9%, and Grippotyphosa 6.3%. Overall prevalence of Hardjo seropositivity found by Otte and colleagues was therefore much higher than those found here for cattle from unvaccinated herds (19.3%), while minor differences were noted for the other serovars. However, their findings cannot be easily compared to our results, because, in addition to the different location, they used a cutoff point of 50 and performed a convenience sampling both of herds and individual animals.

Cross-reactions between serogroups are as common in humans as in animals [23,33] and it is a common notion that the highest MAT titer occurs against the infecting serovar. However, the value of MAT as a predictor for determining the infecting serovar or serogroup is supposedly very low [33,34], although a high predictive value was also reported [35]. In some cases, even a fourfold rising of MAT titer could not allow a certain determination of the infecting serovar in individual patients [36]. The possibility of coinfection with multiple serovars, especially in areas of high endemicity, and paradoxical reactions, which may occur in up to 50% of cases, may be also present and act as confounding factors [37,38,39]. For reliable information on infecting serovars and serogroups, isolation and serological characterization of leptospires is required. Therefore, the breakdown of the serogroup with the highest titer for each cow/herd was not determined.

Triangle 9 vaccine, in addition to BPIV-3, BoHV-1, BVDV, and BRSV, contains five inactivated *Leptospira* serovars, namely Canicola, Grippotyphosa, Hardjo, Icterohaemorrhagiae, and Pomona. Twenty out of 248 seropositive animals from vaccinated herds had high titers, between 1000 and 4000, for one or more serovars. In large animals, vaccine titers are seldom higher than 1:800 and typically vaccinated animals have titer elevation for only the serovars found in the vaccine [1]. Therefore, we could speculate that the high titers found and the presence of a high rate of abortions in the herd could be indicative of infection [14]. However, we do not have data that support evidence of infection or a causal relationship between the high antibody titers found in some animals and abortions. Serovars responsible for titers ≥1000 in animals from vaccinated herd were mainly Hardjo, Pomona, and Canicola. Only one of these animals was positive for the serovar Copenhageni, with a titer equal to 4000. Lower titers were found for Bratislava and Tarassovi. Therefore, positivities with titer ≥1000 in animals from the vaccinated herd were all against serovars or serogroups included in the vaccine. Immunity should not be estimated based on MAT titers in vaccinated animals as protection against clinical disease may be present with very low titers [26]. However, 37.7% of animals belonging to the vaccinated herd were seronegative and a failure of vaccination immunity or the presence of unvaccinated animals cannot be excluded. The vaccinated herd was also the one with the highest rate of abortions, but this data could be biased by poor recording of anamnestic data in some of the farms.

Regarding zoonotic aspects, human leptospirosis seroprevalence reported for other areas of Colombia was high [7,9]. However, here, as in Europe [40], the most common serotypes involved in human disease were Icterohaemorrhagiae and Grippotyphosa. The main reservoir species for serogroup Icterohaemorrhagiae worldwide is generally represented by rats [14,40]. In Europe, namely in France, serovar Grippotyphosa was found mostly in voles and other small rodents [40]. However, in countries where skunks, raccoons, and opossums are present, as in the American continent, they can also play a role as maintenance hosts [41]. Our results showed that serovars Grippotyphosa and Copenhageni, the latter belonging to the Icterohaemorrhagiae serogroup, are among those with the lowest seroprevalences. This suggests that cattle could be a minor risk factor for the transmission of *Leptospira* infection to humans in the sampled area.

The dairy area of Boyacá, together with the Savannah of Bogotá, is responsible for much of the national milk production and is characterized by being a region with prevalence of small- and medium-sized dairy farms. Boyacá does not constitute a continuous plateau, but rather is characterized by a series of very fertile valleys that alternate with mountains. The critical point in the dairy chain of this area is livestock management within the herd, since appropriate hygienic standards are lacking [42]. Moreover, the majority of cattle farms raise several breeds or crossbreeds, without effective production strategies based on animal selection. Also, biosecurity measures are unsatisfactory and the presence of other domestic animals or pets is frequent in cattle herds of this area. The breeding of livestock belonging to different animal species may also be present. Due to sociocultural characteristics, the presence of dogs in direct contact with cattle is very common. Dogs were present in all the examined herds. Moreover, farms workers usually keep poultry for their own consumption, thus promoting the presence of rodents. Due to free grazing, cattle can easily come in contact with other wild animals, in addition to rodents, that can potentially transmit the infection [41]. Rodents are frequently carriers of leptospires of the serotype Icterohaemorrhagiae. However, we found no positives to the Icterohaemorrhagiae–Copenhageni strain among unvaccinated animals. This suggests at least a nondominant epidemiological importance of rodents in the spread of the disease in the tested herds. However, the relation between the presence of other domestic animals and/or wild animals and *Leptospira* seroprevalence in cattle was not evaluated, as the study was underpowered with respect to doing an appropriate statistical analysis.

## 5. Conclusions

In the present study, a high proportion (95%) of herds with at least one MAT-positive animal was found. Moreover, within-herd seroprevalence was very high in 20% of the unvaccinated herds. The study area was characterized by a very high prevalence (97%) of small herds. The presence of different species of other domestic animals on farms, which occurs very frequently on dairy cattle farms in Colombia, and the frequent lack of appropriate hygienic standards could be a risk factor for the spread of *Leptospira* infection among unvaccinated cattle in the considered area.

## Figures and Tables

**Table 1:** Herd-level descriptive data on a convenience sample of 21 dairy herds in Boyacá department (Columbia) that were tested for antibodies to 7 *Leptospira* serovars using microscopic agglutination test.

Herd	Municipality	N. of Animals Tested/Total	Vaccination against *Leptospira* spp.	Presence of Abortions	Presence of other Animal Species ^a^	Milking Mode	Herd Modernization
1	Soracá	23/23	NO	YES	C, D, H	manual	low
2	Ventaquemada	13/13	NO	YES	D	mechanical	medium
3	Ventaquemada	22/22	NO	NO	D	mechanical	medium
4	Ventaquemada	27/27	NO	NO	C, D	mechanical	medium
5	Ventaquemada	21/21	NO	NO	D	mechanical	medium
6	Oicatá	31/31	NO	NO	D	mechanical	medium
7	Oicatá	31/31	NO	NO	C, D, H	mechanical	medium
8	Oicatá	38/38	NO	YES	D	mechanical	medium
9	Oicatá	63/63	NO	YES	D, Po	mechanical	medium
10	Oicatá	31/31	NO	YES	D	mechanical	medium
11	Sopó	398/700	YES	YES (high)	C, D, H	mechanical	high
12	Chiquiza	64/64	NO	NO	D	manual	low
13	Oicatá	40/40	NO	YES	D, Pi	manual	low
14	Oicatá	11/11	NO	YES	D	mechanical	medium
15	Oicatá	9/9	NO	YES	D	mechanical	medium
16	Paipa	21/21	NO	NO	D	manual	low
17	Paipa	25/25	NO	YES	D	mechanical	medium
18	Sotaquira	22/22	NO	YES	D, Po	mechanical	medium
19	Tuta	25/25	NO	NO	C, D, H	mechanical	medium
20	Oicatá	18/18	NO	NO	D	manual	low
21	Oicatá	26/26	NO	NO	C, D, H	mechanical	medium

^a^ C = cats, D = dogs, H = horses, Pi = pigs; Po = poultry.

**Table 2:** Overall seroprevalence to 7 *Leptospira* serovar in cattle from 20 unvaccinated dairy herds and one vaccinated dairy herd in Boyacá department (Columbia) that were tested using microscopic agglutination test.

Serovar	Animals from Unvaccinated Herds Pos/All Animals (%)	Animals from Vaccinated Herd Pos/All Animals (%)	Animals from all Herds Pos/All Animals (%)
Bratislava	2/561 (0.4)	34/398 (8.5)	36/959 (3.8)
Canicola	28/561 (5.0)	204/398 (51.3)	232/959 (24.2)
Copenhageni	0/561 (0)	28/398 (7.0)	28/959 (2.9)
Grippotyphosa	5/561 (0.9)	17/398 (4.3)	22/959 (2.3)
Hardjo	108/561 (19.3)	228/398 (57.3)	336/959 (35.0)
Pomona	17/561 (3.0)	144/398 (36.2)	161/959 (16.8)
Tarassovi	2/561 (0.4)	57/398 (14.3)	59/959 (6.2)

**Table 3:** Animals from 20 unvaccinated dairy herds and one vaccinated dairy herd in Boyacá department (Columbia) that showed single or multiple positivity to different *Leptospira* serovars on the microscopic agglutination test.

Number of Serovars to Which the Animal Is Positive	Animals from Unvaccinated Herds Animals/All Positive Animals (%)	Animals from Vaccinated Herd Animals/All Positive Animals (%)	Animals from all Herds Animals/All Positive Animals (%)
**1**	121/135 (89.6)	42/248 (16.9)	163/383 (42.6)
**2**	7/135 (5.2)	53/248 (21.4)	60/383 (15.7)
**3**	3/135 (2.2)	95/248 (38.3)	98/383 (25.6)
**4**	2/135 (1.5)	23/248 (9.3)	25/383 (6.5)
**5**	2/135 (1.5)	25/248 (10.1)	27/383 (7.0)
**6**	0/135 (0)	8/248 (3.2)	8/383 (2.1)
**7**	0/135 (0)	2/248 (0.8)	2/383 (0.5)

**Table 4:** Within-herd *Leptospira* seroprevalence in animals from 20 unvaccinated dairy herds and one vaccinated (herd n. 11) dairy herd in Boyacá department (Columbia) that were tested for antibodies to 7 *Leptospira* serovars using microscopic agglutination test.

Herd	AB ^a^	C	IC	G	H	P	T	Total
1	0/23 (0) ^b^	3/23 (13.0)	0/23 (0)	0/23 (0)	18/23 (78.3)	0/23 (0)	0/23 (0)	18/23 (78.3)
2	0/13 (0)	0/13 (0)	0/13 (0)	0/13 (0)	9/13 (69.2)	0/13 (0)	0/13 (0)	9/13 (69.2)
3	0/22 (0)	0/22 (0)	0/22 (0)	0/22 (0)	4/22 (18.2)	0/22 (0)	0/22 (0)	4/22 (18.2)
4	0/27 (0)	0/27 (0)	0/27 (0)	0/27 (0)	5/27 (18.5)	0/27 (0)	0/27 (0)	5/27 (18.5)
5	0/21 (0)	0/21 (0)	0/21 (0)	0/21 (0)	14/21 (66.7)	0/21 (0)	0/21 (0)	14/21 (66.7)
6	0/31 (0)	2/31 (6.5)	0/31 (0)	0/31 (0)	5/31 (16.1)	0/31 (0)	0/31 (0)	7/31 (22.6)
7	0/31 (0)	1/31 (3.2)	0/31 (0)	0/31 (0)	25/31 (80.6)	0/31 (0)	0/31 (0)	26/31 (83.9)
8	0/38 (0)	2/38 (5.3)	0/38 (0)	0/38 (0)	0/38 (0)	0/38 (0)	0/38 (0)	2/38 (5.3)
9	0/63 (0)	2/63 (3.2)	0/63 (0)	0/63 (0)	1/63 (1.6)	2/63 (3.2)	0/63 (0)	3/63 (4.8)
10	1/31 (3.2)	3/31 (9.7)	0/31 (0)	0/31 (0)	0/31 (0)	0/31 (0)	0/31 (0)	4/31 (12.9)
11	34/398 (8.5)	204/398 (51.3)	28/398 (7.0)	17/398 (4.3)	228/398 (57.3)	144//398 (36.2)	57/398 (14.3)	248/398 (62.3)
	[6.7–10.3] ^c^	[48–54.6]	[5.3–8.7]	[3–5.6]	[54–60.6]	[33–39.4]	[12–16.6]	[59.1–65.5]
12	0/64 (0)	3/64 (4.7)	0/64 (0)	0/64 (0)	4/64 (6.3)	2/64 (3.1)	0/64 (0)	/64 (10.9)
13	0/40 (0)	1/40 (2.5)	0/40 (0)	0/40 (0)	7/40 (17.5)	3/40 (7.5)	0/40 (0)	9/40 (22.5)
14	0/11 (0)	1/11 (9.1)	0/11 (0)	0/11 (0)	0/11 (0)	0/11 (0)	0/11 (0)	1/11 (9.1)
15	0/9 (0)	0/9 (0)	0/9 (0)	0/9 (0)	0/9 (0)	0/9 (0)	0/9 (0)	0/9 (0)
16	0/21 (0)	1/21 (4.8)	0/21 (0)	0/21 (0)	0/21 (0)	0/21 (0)	0/21 (0)	1/21 (4.8)
17	0/25 (0)	4/25 (16.0)	0/25 (0)	5/25 (20.0)	9/25 (36.0)	7/25 (28.0)	2/25 (8.0)	12/25 (48.0)
18	1/22 (4.5)	1/22 (4.5)	0/22 (0)	0/22 (0)	0/22 (0)	1/22 (4.5)	0/22 (0)	3/22 (13.6)
19	0/25 (0)	1/25 (4.0)	0/25 (0)	0/25 (0)	0/25 (0)	0/25 (0)	0/25 (0)	1/25 (4.0)
20	0/18 (0)	0/18 (0)	0/18 (0)	0/18 (0)	1/18 (5.6)	1/18 (5.6)	0/18 (0)	2/18 (11.1)
21	0/26 (0)	3/26 (11.5)	0/26 (0)	0/26 (0)	6/26 (23.1)	1/26 (3.8)	0/26 (0)	7/26 (26.9)

^a^ AB = Bratislava, C = Canicola, IC = Copenhageni, G = Grippotyphosa, H = Hardjo, P = Pomona, T = Tarassovi, ^b^ positive animals/all animals (% seroprevalence); ^c^ 95% confidence interval.
